# Smart windows based on VO_2_ and WS_2_ monolayers

**DOI:** 10.1039/d5na00584a

**Published:** 2025-07-23

**Authors:** Mahdieh Hashemi, Mona Gandomi, Maryam Moradi, Narges Ansari

**Affiliations:** a Department of Physics, College of Science, Fasa University Fasa 74617-81189 Iran mahdieh.hashemi@gmail.com; b Department of Atomic and Molecular Physics, Faculty of Physics, Alzahra University Tehran 1993893973 Iran

## Abstract

Smart windows automatically adjust their properties to control infrared (IR) radiation which helps with saving energy by reducing the need for heating and cooling. In the current study, we use vanadium dioxide (VO_2_), a phase change material that in temperatures above 68 °C behaves as a metal, to design smart windows. In its metallic phase, VO_2_ transmits less IR than would be expected from a smart window. Visible light transmission through the VO_2_ window in both its insulator and metallic phase is low which causes low indoor lighting. To solve this problem, we propose structuring the VO_2_ as a grating on a silica substrate. A thin film of VO_2_ blocks 62% of IR and transmits 46% of visible light, while a grating with an 800 nm period and 700 nm ribbon width improves IR blockage to 67%/63% and increases the visible light transmission to 53%/47% under transverse magnetic (TM)/electric (TE) radiation. Another issue of VO_2_ windows is the unpleasant yellow-brownish color of them. To solve this problem, we inserted atomic layers of Tungsten disulfide (WS_2_) in the window structure. Adding 5 layers of WS_2_ keeps IR blockage and visible light transmission almost the same, while reducing transmission at the wavelength of 620 nm from 49%/41% in the case of VO_2_ grating, to 34%/30% under TM/TE radiation, which changes the window color. These window properties are consistent under different light angles. Notably, in the proposed VO_2_-based smart windows, all three critical factors of visible light transmission, IR blockage, and pleasant window color are simultaneously optimized for an unpolarized incident light in a wide range of radiation angles. The simulated reported results of this paper pave a new way in the world of smart windows.

## Abstract 2

Smart windows are designed based on VO_2_ and WS_2_ monolayers, with all three critical factors of visible light transmission, IR blockage, and pleasant color simultaneously optimized for an unpolarized incident light in a wide range of radiation angles.

## Graphical abstract

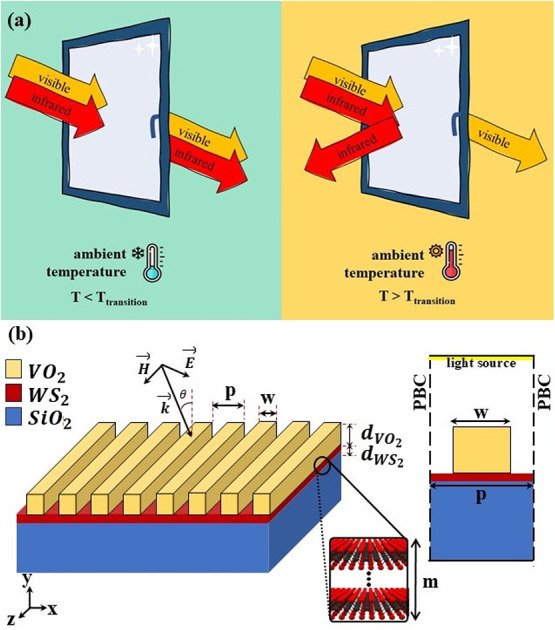

## 1 Introduction

The issue of global warming makes it a necessity to construct green housing and conserve the building’s energy properly.^1^ To wisely manage the energy usage in the buildings, smart architectural designs are highly preferable.^2^ As most of the heat exchange between the interior and outside of the buildings occurs through the windows, smart windows are mostly recommended in the context of energy conservation.^3–5^ Generally, smart designs are categorized into two types: active and passive ones.^6,7^ Within the active designs inclusion of an external power supply like an external voltage or magnet is required.^8–10^ In contrast, passive designs automatically adjust their functionality to adapt the environmental conditions.^11,12^ To reduce our demand for fossil fuels for supplying any external energy source, passive designs are advantageous. In designing passive smart windows, thermochromic and photochoromic materials that change their optical properties under temperature and light are mostly studied.^13,14^ By using such materials in windows, reflection and transmission of the incoming light and specifically the infrared (IR) radiation can be tuned. By controlling the IR radiation, both heat gain and loss would be modulated passively to regulate the internal temperature. This way, the smart windows should block the IR radiation from entering the house in hot weather, while letting it pass in cold conditions.^15^

Vanadium dioxide (VO_2_) is one of the famous thermochromic materials that undergoes a phase transition from insulator to metallic state^16,17^ when the temperature exceeds a critical temperature of 68 °C.^18^ At temperatures below 68 °C, VO_2_ is a monoclinic narrow band gap semiconductor with a gap of 0.7 eV that is transparent under IR radiation.^19^ Above this temperature, in its tetragonal metallic phase, IR radiation would be blocked. However, if the windows were manufactured purely from VO_2_, we would face two other issues: low visible light transmission in both the semiconductor and metallic state of VO_2_ (ref. 17, 20 and 21) and an unfavorable brown color.^17,22,23^ The challenges with VO_2_ are how to modify this brownish color, while keeping the visible light transmission as high as possible. It should be considered that together with these challenges, in any possible VO_2_-based structure that is designed to be used in smart window applications, IR blockage also should not be reduced. Several works have attempted to improve the performance of VO_2_-based smart windows by increasing the visible light transmission,^24^ tuning the IR transparency,^25–27^ adjusting the window color,^17,23,28–31^ and reducing the critical transition temperature of VO_2_ from its intrinsic value of 68 °C.^32–34^ Since typical ambient temperatures do not reach this transition point, practical application of VO_2_ smart windows requires lowering the phase transition temperature. This can be achieved through methods such as adding dopants,^33,35–37^ utilizing multilayer structures,^34,38^ and inserting nanoparticles.^39,40^ But the trade-off between improving one parameter and missing another makes it difficult to design a window that is perfect.

In the current study, we optimize all three parameters of the smart VO_2_-based windows together: increasing the visible light transmission, more IR blockage in hot weather, and changing the window color under illumination with an unpolarized light and with different incident angles. This has been done by structuring the VO_2_ as a nanometer-sized grating on a silica (SiO_2_) substrate with usage of monolayers of WS_2_ for controlling the visual color of the window. WS_2_ is one of the two-dimensional transition metal dichalcogenides (TMDCs) with three excitonic absorption peaks in the visible spectrum with wavelengths around 443 nm, 517 nm, and 620 nm.^41^ This way, WS_2_ with these absorption peaks at the visible spectrum is a perfect candidate for manipulating the window color, while due to its atomic thickness, the window remains nanometer-sized.^42^ Compared to the other TMDCs, other than strong excitonic absorption in the visible range, WS_2_ is advantageous in photonic applications due to its notable photoresponsivity, chemical stability, and excellent photoresponse time.^43–45^ In practice, the WS_2_ atomic-layers can be synthesized using various techniques such as chemical vapor deposition, spin-coating of solution-processed precursors, or sulfurization of pre-deposited tungsten-containing films. These methods allow for control over the number of layers and film uniformity, as demonstrated in previous studies.^46,47^ Thickness characterization can be achieved using Raman spectroscopy and atomic force microscopy.^48^ The VO_2_ gratings can be made by combining standard thin-film deposition techniques (such as pulsed laser deposition or sputtering) with high-resolution patterning methods like electron-beam lithography and reactive ion etching.^49^

Throughout the manuscript, we try to optimize our smart window properties by changing the geometrical parameters of the grating, period and width of the ribbons, together with the number of WS_2_ atomic layers in the structure. As sun light is not polarized, we examine the two linear orthogonal polarizations of the incident light, transverse magnetic (TM) and transverse electric (TE), and optimize the geometrical parameters of the designed window for both polarizations. Moreover, as sunlight can strike surfaces from a wide range of angles, we also evaluate our optimized structure under different illumination angles. Ultimately, we indicate that the optimized structure exhibits acceptably robust performance across both polarizations and a wide range of incident angles, confirming its practical stability under real sunlight conditions.

## 2 Smart window design and simulation method

**Fig. 1:**
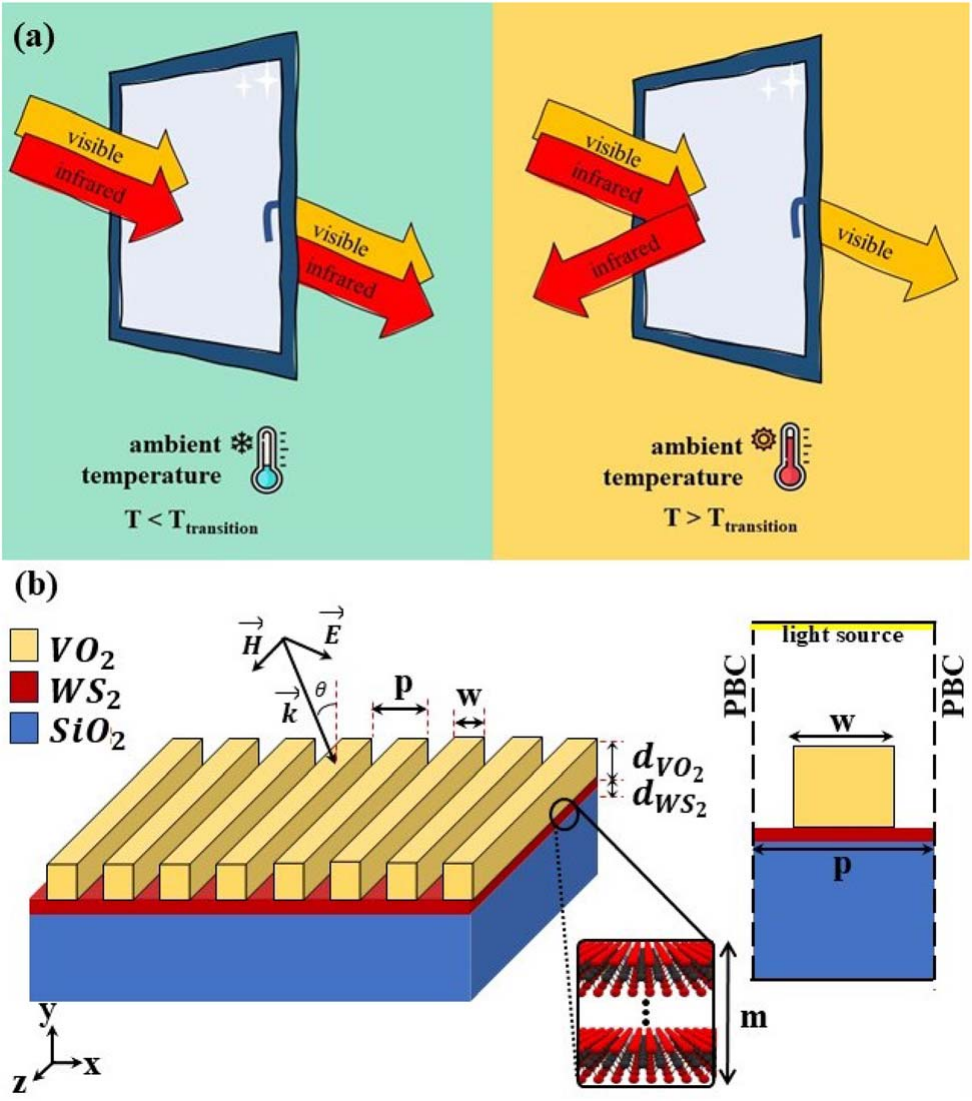
(a) A general schematic of the smart windows functionality under the illumination of visible and IR radiation. (b) Schematic of our proposed smart window structure with VO_2_ grating on a silica substrate with the WS_2_ monolayers inserted between them. It also includes a schematic of the unit cell of the designed grating with the utilized boundary conditions.

Fig. 1a shows a schematic of thermochromic smart windows that allow transmission of visible light both below and above the critical temperature, while the IR radiation transmits through it in cold weather and blocks in hot weather. In Fig. 1b our designed VO_2_-based smart window is shown with the VO_2_ grating placed on a silica substrate. WS_2_ monolayers are inserted between the grating and substrate. The period of the grating is denoted by *p*, and the width and height of the ribbons are labeled by *w* and *d*_VO_2__, respectively. Thickness of the WS_2_ layer is assigned by *d*_WS_2__ which consisted of *m* layers of WS_2_ monolayer each with a thickness of 0.61 nm.^41^

As shown in Fig. 1b, the incidence angle of the illuminating light relative to the grating’s normal is denoted by *θ*. The incident light’s electric field (*E*), magnetic field (*H*), and wave vector (*k*) form a right-handed coordinate system. When the *H*-field is parallel to the ribbons (which are perpendicular to the incident plane, denoted as *H*_*z*_), the *E*-field lies within the incident plane, corresponding to transverse magnetic (TM) polarization. Conversely, for transverse electric (TE) polarization, the *E*-field is perpendicular to the incident plane (*E*_*z*_).

Since the VO_2_ ribbons are arranged on the substrate and extend infinitely in the direction perpendicular to the incident plane, a two-dimensional configuration is sufficient for simulation. To illustrate the simulation method used, Fig. 1b also includes a schematic of the unit cell of the designed grating. The unit cell is enclosed by periodic boundary conditions in the *x*-direction, allowing it to replicate and form the full grating. As shown in the figure, the upper boundary is defined as the light source, while the lower boundary, which is connected to the silica substrate, is set as a perfectly matched layer (PML) to simulate the infinite extension of the substrate. Given that WS_2_ is an atomically thin layer, to avoid meshing issues and excessive computational load, it is modeled as a boundary with a thickness of *m* × *d*_WS_2__.

The optical constant of both VO_2_ and WS_2_ follows the Drude–Lorentz formula as eqn (1):1
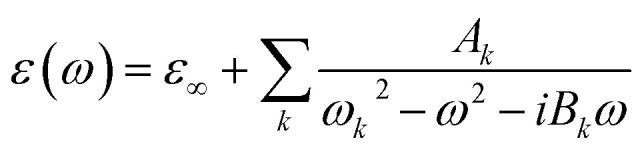
in which *ω* is the angular frequency of the incident light, *ω*_*k*_ and *B*_*k*_ are the angular frequency and the damping constant of the *k*th resonant interband transition, respectively.

For *d*_VO_2__ = 50 nm, that we kept fixed throughout the paper, *ε*_∞_ = 4.0 in the insulator phase of VO_2_ and *ε*_∞_ = 4.77 in its metallic phase. Other constant parameters of eqn (1) for both the metallic and insulator phases of VO_2_ up to the first three resonances, the *A*_*k*_s and *B*_*k*_s with *k* ≤ 3, are taken from ref. 20. For WS_2_ the *ε*_∞_ is 7.449 and constant *A*_*k*_ and *B*_*k*_ values up to the forth resonance are taken from ref. 50–52. It should be noted that figures in the paper, except Fig. 4, are all plotted under TM polarized radiation. The results under TE polarization are reported numerically throughout the manuscript.

## 3 Results and discussion

**Fig. 2:**
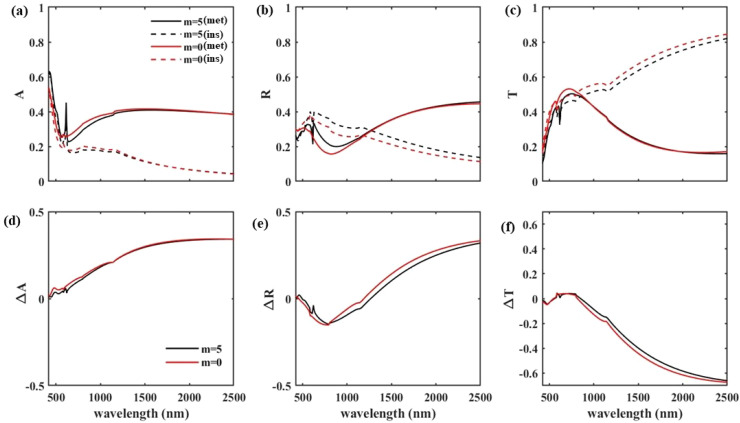
(a–c) Absorption, reflection, and transmission spectra, respectively, of the structure with *d*_VO_2__ = 50 nm, *p* = 800 nm and *w* = 700 nm under normal TM illumination (*θ* = 0): solid lines are used for the metallic and dashed lines for the insulating state of VO_2_, while the red lines are for the case of *m* = 0 and black lines are for *m* = 5. (d–f) The difference between the absorption, reflection, and transmission when the VO_2_ changes its phase from metallic to insulator with red lines for the case of *m* = 0 and black lines for *m* = 5.

To show the optical performance of our proposed window, the absorption (*A*), reflection (*R*), and transmission (*T*) spectra of the structure with *p* = 800 nm and *w* = 700 nm under normal TM illumination (*θ* = 0), with and without WS_2_ monolayers are included in Fig. 2a–c, respectively. In these figures, the red solid and dashed lines are representative of *A*, *R*, and *T* of the structure without WS_2_, *m* = 0, when the VO_2_ is in its insulator and metallic state, respectively. It can be seen that, without WS_2_, visible light transmission more than 50% at the wavelength of 700 nm in both insulator and metallic states of VO_2_ is reached. Under TE illumination this 50% for TM radiation reaches 47% for the same structure with *p* = 800 nm, *w* = 700 nm, and *m* = 0. This visible light transmission is one of the advantages of our designed window in which both in the cold and hot weather more than half of the visible light passes through the window and lighten the interior of the building. In contrast, a drastic decrease of IR transmission can be recognized in Fig. 2c with change of VO_2_ state from insulator to metallic, which is essential for heat filtering in hot weather and is expected from a smart window. To have a better insight into changes in light absorption, reflection, and transmission through the change of the VO_2_ state from metallic (above the critical temperature of 68 °C) to insulator (bellow the critical temperature) in Fig. 2d–f we included spectrum of Δ*A*, Δ*R*, and Δ*T*, respectively. In all figures the red lines show the resulting Δ*A*, Δ*R*, and Δ*T* in the structure without WS_2_. It can be seen that, at the wavelength of 2500 nm, Δ*A* reaches 34%, Δ*R* becomes 33%, and Δ*T* shows a significant value of 67% that is a guarantee for the smart window to filter the incoming heat and perform efficiently.

With inclusion of 5 layers of WS_2_, Δ*A*, Δ*R*, and Δ*T* of the structure are shown in Fig. 2d–f by black lines. By considering the minute difference between the observed Δ*A*, Δ*R*, and Δ*T* of the structure with and without WS_2_, fortunately the window’s IR blockage and visible light transmission would not be affected by WS_2_ inclusion. Focusing on Fig. 2a–c clarifies the role of WS_2_ layers in the structure. The *A*, *R*, and *T* of the structure with five layers of WS_2_, *m* = 5, are shown by black solid and dashed lines when the VO_2_ is in its metallic and insulator state, respectively. In Fig. 2a at the wavelength of 620 nm, the wavelength of one of the WS_2_ bandgaps, an abrupt increase in light absorption occurs in the structure. Within the metallic phase of VO_2_, solid lines, light absorption is increased from 25% in structure without WS_2_ to 45% in the structure with WS_2_. Equivalently, in this structure insertion of WS_2_ causes reduction of light transmission from 49% to 34% (Δ*T* = 15%). In the insulator phase of VO_2_, dashed lines, the absorption increases from 18% in the structure without WS_2_ to 40% within the structure with 5 layers of WS_2_, *i.e.* along with the same value of Δ*T* = 15% like the metallic structure. For TE illumination, in the metallic state of VO_2_, light transmission at *λ* = 620 nm decreases from 41% to 30% with insertion of 5 layers of WS_2_ that is equivalent to Δ*T* = 11%. Within the insulator phase of VO_2_, the Δ*T* value reaches 8%. This change in light transmission, at the visible wavelength range, affects the window’s color and helps fix the concerns around the unpleasant color of the window. It should be mentioned that, although the inclusion of monolayers of WS_2_ reduces the visible light transmission around *λ* = 620 nm it gives us a better window color without any unwanted effect on increasing the window thickness or modifying the IR blockage.

**Fig. 3:**
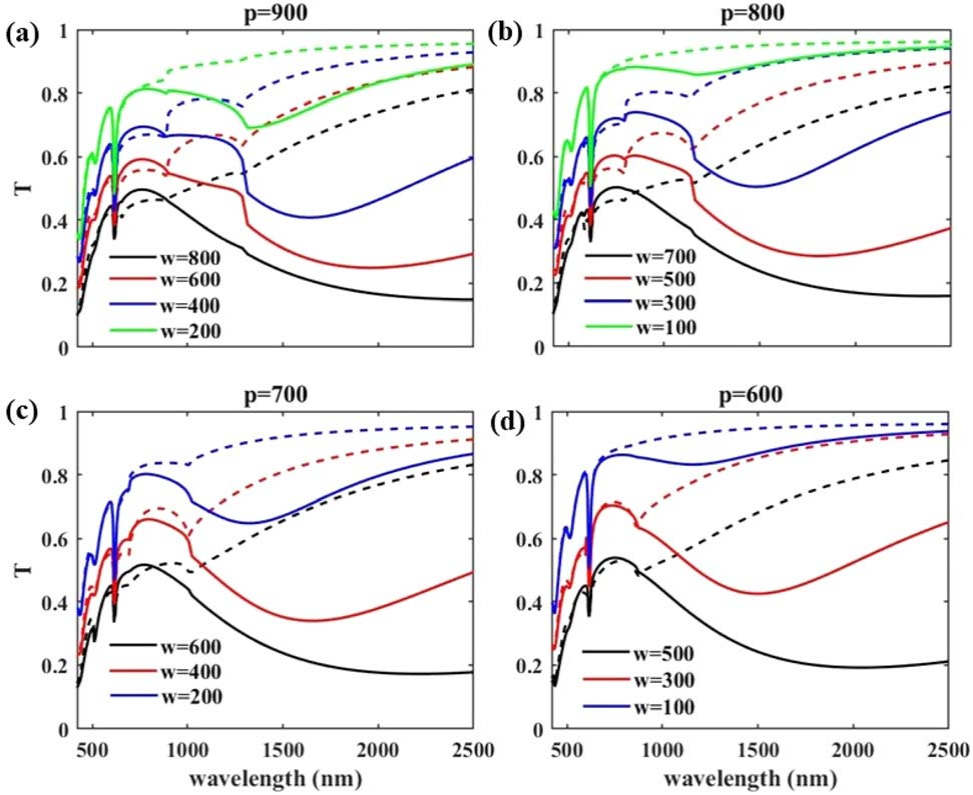
Transmission spectra of the structures with (a) *p* = 900 nm, (b) *p* = 800 nm, (c) *p* = 700 nm, and (d) *p* = 600 nm with different values of *w* swept in steps of 200 nm under TM illumination. Other structure parameters are kept fixed as *d*_VO_2__ = 50 nm, *m* = 5, and *θ* = 0. Solid lines are for the metallic and dashed lines for the insulating state of VO_2_.

In our exploration of optimizing the window efficiency, in Fig. 3 we represent the transmission spectrum of the structures while sweeping over two of the geometrical parameters of the structure: *p* and *w*, and keeping fixed *d*_VO_2__ = 50 nm, *m* = 5, and *θ* = 0. In Fig. 3a–d transmission spectra of the structures with *p* = 900 nm, *p* = 800 nm, *p* = 700 nm, and *p* = 600 nm are shown, respectively. In each period, with steps of 200 nm, a sweep over *w* is done and the results are reported by solid/dashed lines for VO_2_ in its metallic/insulator phase. In all figure parts it is obvious that irrespective to the period, with small values of *w* compared to *p*, IR blockage in hot weather has not happened efficiently. While, with larger *w* values, the window blocks IR radiation significantly above the critical temperature but with the cost of reduction in visible light transmission. In all presented structures a sharp dip at the transmitted light at the wavelength of *λ* = 620 nm is apparent. These dips that are characteristic of WS_2_ absorption in the structure, modify the color of our designed windows and appear to be a solution to the unpleasant color of VO_2_ windows.

It is worth mentioning that, to confirm practical feasibility, we examined the sensitivity of our designed smart windows to ± 20 nm variations in *w* and slight changes in *p*. The results showed a negligible impact on performance, indicating that the structure is robust against typical fabrication tolerances.

**Fig. 4:**
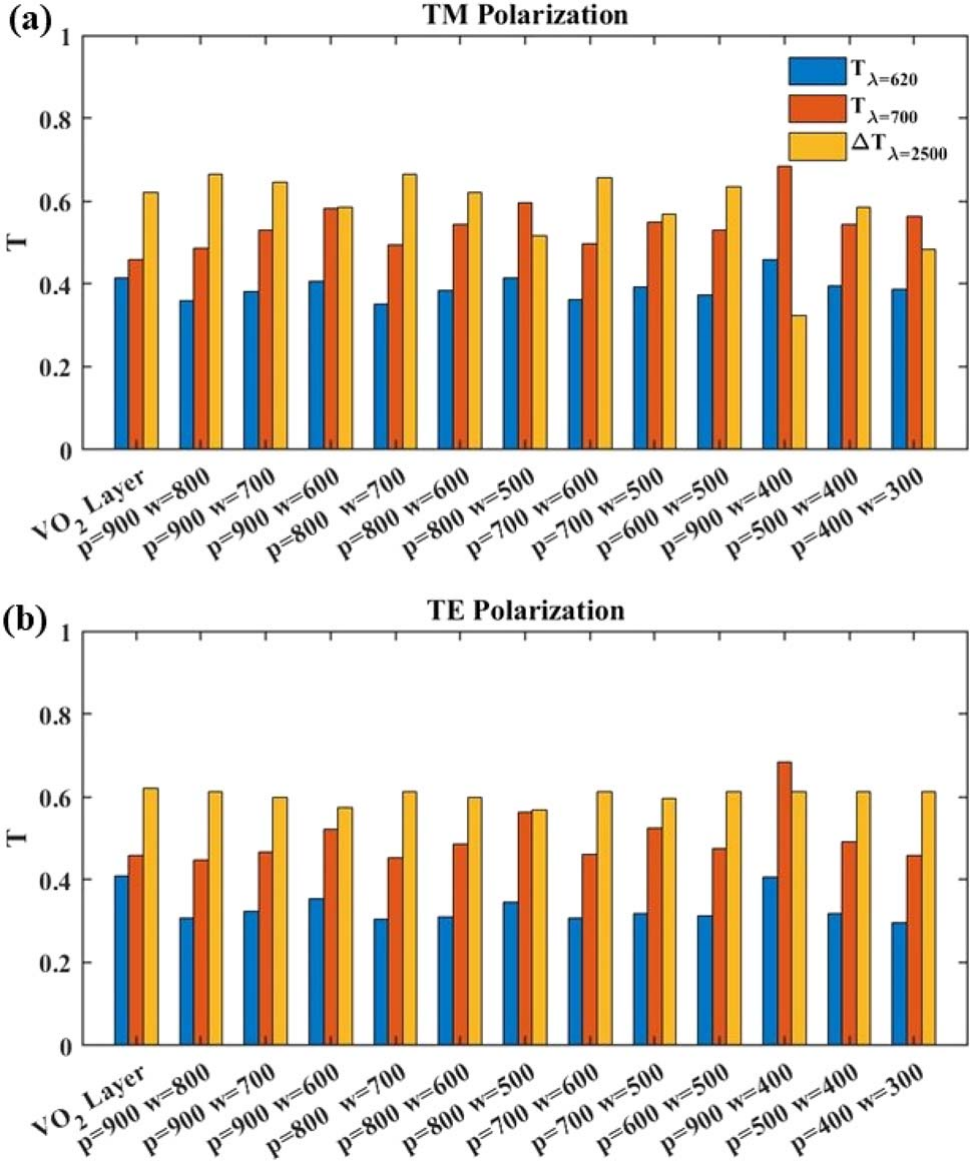
Bar chart of three factors of smart windows: filtration of *λ* = 620 nm with blue bars (*T*_*λ*=620_), visible light transmission with red bars (*T*_*λ*=700_), and yellow bars for IR blockage at *λ* = 2500 nm (Δ*T*_*λ*=2500_), within the structures with different values of *p* and *w* under (a) TM (b) TE polarization illumination. *m* = 5 and *θ* = 0 is fixed for all studied structures.

To make it easier to compare performance of the structures with acceptable results under TM (Fig. 4a) and TE (Fig. 4b) illumination, a bar chart of three important factors of smart windows is presented: filtration of *λ* = 620 nm with blue bars (*T*_*λ*=620_) for pleasant color of the window and visible light transmission with red bars (*T*_*λ*=700_) both in the metallic phase of VO_2_, and yellow bars for IR blockage at *λ* = 2500 nm (Δ*T*_*λ*=2500_), that is the change of transmission value when VO_2_ modifies from its insulator phase to its metallic one. All studied structures have 5 layers of WS_2_ and *θ* is set to 0 in them. To show the advantages of our suggested smart window, bars of a uniform VO_2_ layer in its metallic state with thickness of 50 nm are also included in the figure.

Comparing the blue bars of all structures with that of the uniform VO_2_ layer show that in all structures reduction of light transmission at *λ* = 620 nm occurs. The lowest value of *T*_*λ*=620_ belongs to the structure with *p* = 800 nm and *w* = 700 nm that reduces the light transmission at *λ* = 620 nm from 41% in case of uniform VO_2_ layer to 34% for TM illumination and 30% for TE illumination. The red bars of Fig. 4 show that in the plain VO_2_ layer, *T*_*λ*=700_ has the value of 46%. With structuring the VO_2_ as gratings, in all studied structures *T*_*λ*=700_ increases from that of the VO_2_ layer, and a maximum value of 68% is reached in the structure with *p* = 900 nm and *w* = 400 nm under TM illumination. In case of TE polarization illumination at the same structure geometry, a maximum value of *T*_*λ*=700_ = 68% is reached. Yellow bars of Fig. 4 illustrate that in case of unstructured VO_2_, the value of Δ*T*_*λ*=2500_ is 62% while in structures with *p* = 900 nm and *w* = 800 nm, *p* = 800 nm and *w* = 700 nm, and *p* = 700 nm and *w* = 600 nm more IR blockage happens and Δ*T*_*λ*=2500_ reaches 65% for TM polarized illumination. Within all mentioned geometries, *p* = 900 nm and *w* = 800 nm, *p* = 800 nm and *w* = 700 nm, and *p* = 700 nm and *w* = 600 nm, under TE illumination, *T*_*λ*=2500_ reaches 61%. Interestingly, in all of these structures *p* − *w* is 100 nm. To understand the mechanism behind the observed infrared (IR) transmission reduction, we investigated how variations in the grating period (*p*) and width (*w*) influence the interaction between light and the VO_2_-based structure in its metallic phase. By investigating the *H*_*z*_ field distribution, we found that when the difference between the period and width (*p* − *w*) is 100 nm, the incident TM-polarized light at *λ* = 2500 nm is primarily absorbed at the VO_2_/WS_2_/silica interface which enhances absorption that leads to a more pronounced reduction in IR transmission. In contrast, for configurations where *p* − *w* is larger, the light tends to couple into surface waves in the silica/WS_2_/air interface, resulting in less effective attenuation in transmission.

To have a sense of the window color, we used the CIE color system that is a standardized model developed by the International Commission on Illumination (CIE) to quantitatively describe and compare colors based on human vision. In this system, any visible color can be represented as a combination of three parameters: *X*, *Y*, and *Z*. The *Y* component represents luminance (brightness), while *X* and *Z* carry the chromatic information. The *X* is roughly related to the red-green response and the *Z* corresponds mainly to the blue response of the human eye. Based on *X*, *Y*, and *Z* the chromaticity coordinates, *x*, *y* and *z*, are defined with the formulas of eqn (2):^53^2
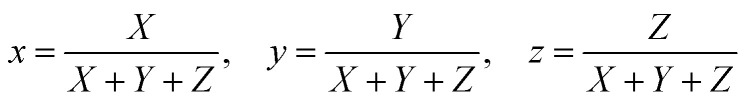


**Fig. 5:**
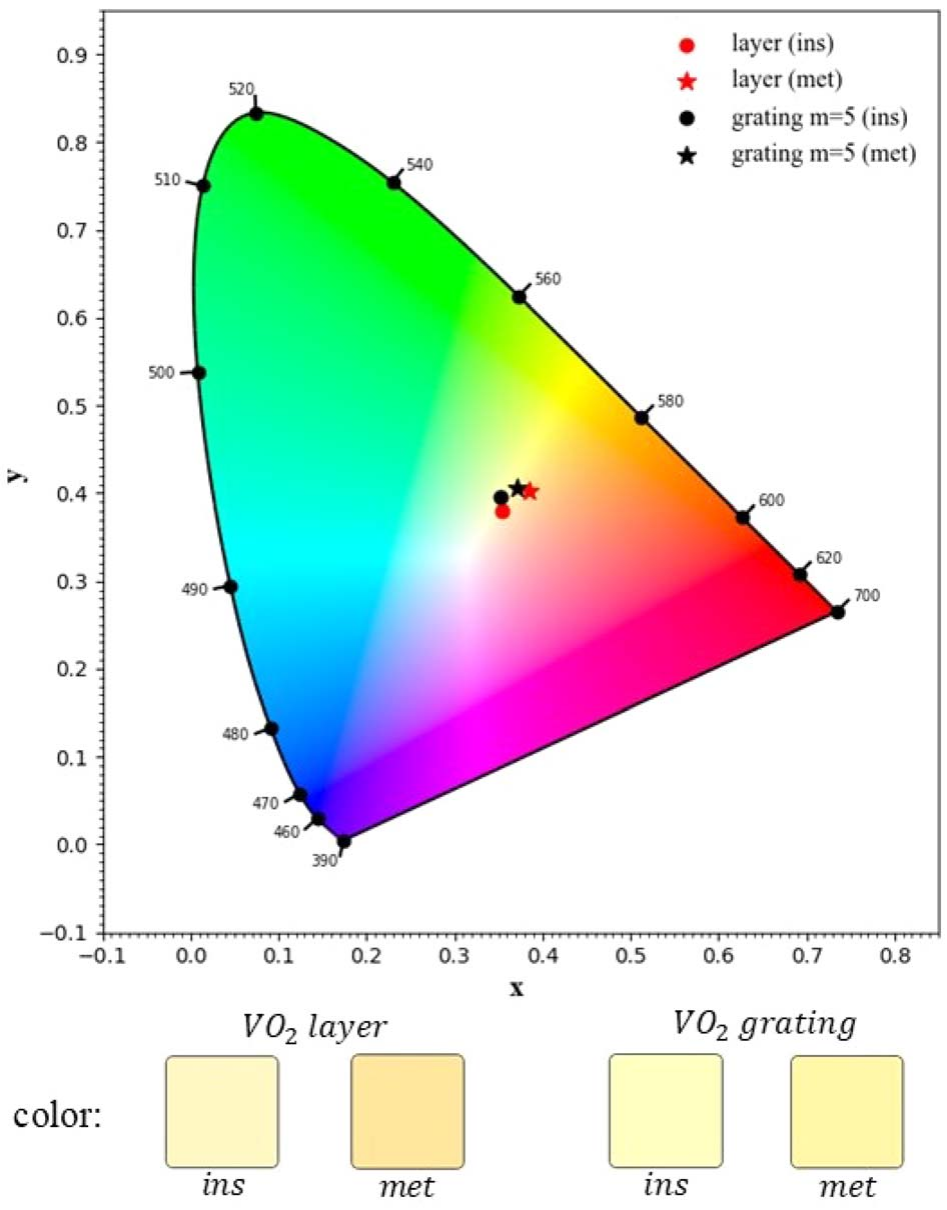
CIE chromaticity diagram of our designed smart window under TM polarization illumination, with (red points) and without (black points) inclusion of WS_2_, and with the VO_2_ phase as metallic (star points) and insulator (circle points). The perceived colors are shown below the graph.

Since *x* + *y* + *z* = 1, only two values are needed to specify a color’s chromaticity; these are *x* and *y* which have also been used in previous studies that visualized the color of VO_2_-based windows.^54–56^ In Fig. 5 we plotted the CIE chromaticity diagram as a function of *x* and *y* where each point represents the perceived color for the average human eye under standard lighting conditions. The red points indicate the perceived color of a 50 nm thick VO_2_ with star for the metallic state of VO_2_ and circles for its insulator phase. The black points indicate the perceived color of our designed grating-type window within metallic (black star) and insulator (black circle) state of VO_2_ with inclusion of 5 layers of WS_2_. To clarify the effect of our designed window on the sensed color by the human eyes, below the graph we added VO_2_ layer colors and our designed window colors in both insulator and metallic states of VO_2_. It is evident that, compared to the plain VO_2_ layer, our designed window reduces the brownish color and results in a more visually appealing perceived color in both states of VO_2_.

To find the best design as a smart window to have optimum visible light transmission and IR blockage with a pleasant color, we need to balance between these three items. As an example, although in the structure with *p* = 900 nm and *w* = 400 nm, *T*_*λ*=700_ reaches the significant value of 68%, its IR blockage is under that of the uniform VO_2_ layer. Careful investigation of the resultant three window parameters show that the structures with *p* − *w* = 100 nm not only have the best IR blockage but also other window parameters in them reach acceptable values. In Table 1 we summarize the results of *T*_*λ*=620_, *T*_*λ*=700_, and Δ*T*_*λ*=2500_ for the three structures with *p* = 900, *w* = 800 nm; *p* = 800, *w* = 700 nm; and *p* = 700, *w* = 600 nm with and without WS_2_ layers.

**Table 1:** *T*_*λ*=620_, *T*_*λ*=700_, and Δ*T*_*λ*=2500_ for the three structures with *p* = 900, *w* = 800 nm; *p* = 800, *w* = 700 nm; and *p* = 700, *w* = 600 nm with and without WS_2_ layers under TM polarization illumination. *T*_*λ*=620_, *T*_*λ*=700_, and Δ*T*_*λ*=2500_ values for a uniform 50 nm VO_2_ layer are also included

	VO_2_ layer	*p* = 900, *w* = 800 nm		*p* = 800, *w* = 700 nm		*p* = 700, *w* = 600 nm	
		*m* = 0	*m* = 5	*m* = 0	*m* = 5	*m* = 0	*m* = 5
*T*_*λ*=620_	41%	49%	36%	49%	34%	50%	36%
*T*_*λ*=700_	46%	52%	49%	53%	50%	53%	50%
Δ*T*_*λ*=2500_	62%	68%	67%	67%	67%	65%	65%

From Table 1, it can be seen that the window parameters of these three structures when they include 5 layers of WS_2_ are more or less the same. Lower *T*_*λ*=620_ in structures with WS_2_ compared with pristine VO_2_ and grating structures without WS_2_ is obvious. With the greater *T*_*λ*=700_, and Δ*T*_*λ*=2500_, the structures with *p* − *w* = 100 nm are highly advantageous over uniform, unstructured VO_2_ windows. For further studies we select the structure with *p* = 800 nm and *w* = 700 nm.

The data of Table 1 shows that, although presence of WS_2_ do not cause any significant change in IR transmission and Δ*T*_*λ*=2500_ remains unchanged, in the visible spectrum it plays a significant role, specially at *λ* = 620 nm.

**Fig. 6:**
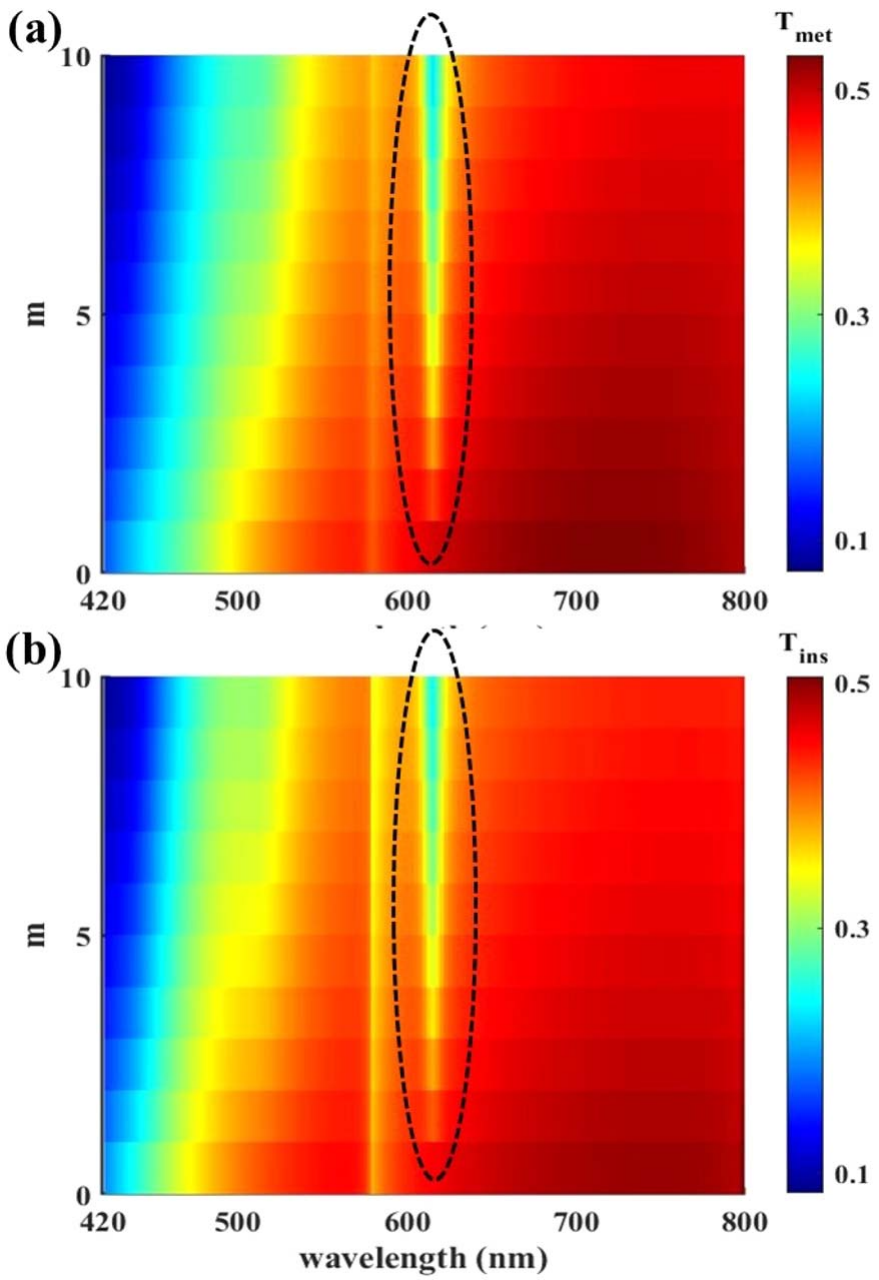
Transmission spectrum of the structure with *p* = 800 nm and *w* = 700 nm for (a) metallic and (b) insulator states of VO_2_ under *θ* = 0 as a function of the number of WS_2_ layers, *m*, under TM polarization illumination. The selected area in both figure’s parts are illustrative of the transmission reduction around the wavelength of 620 nm.

To find the optimum number of WS_2_ layers in the structure, in Fig. 6 we studied the effect of increasing *m* from 0 to 10 on the visible light transmission spectrum of the structure with *p* = 800 nm and *w* = 700 nm for the two states of VO_2_: its metallic state in Fig. 6a and the insulator state of it in Fig. 6b. It can be seen that, in both states of VO_2_, insertion of WS_2_, even only one atomic layer of it, reduces *T*_*λ*=620_ which affects the window color; a solution to the undesirable color of VO_2_ windows. In both Fig. 6a and b the dashed paths indicate the reduction of the transmitted light at *λ* = 620 nm that starts from *m* = 1 and keeps this trend up to the maximum studied layer number of *m* = 10.

**Fig. 7:**
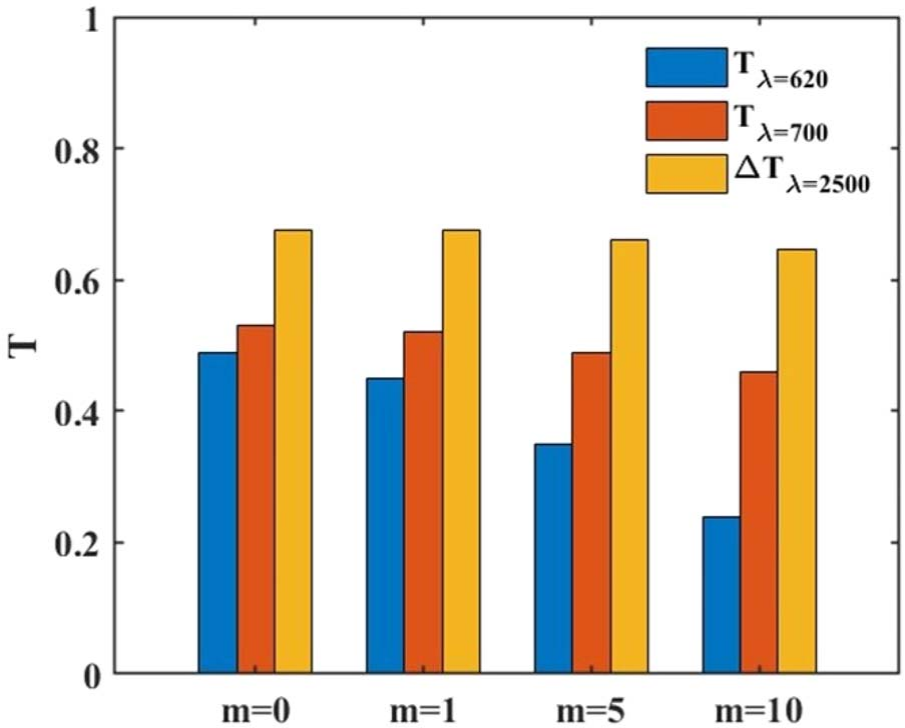
Bar chart of three factors of smart windows under TM polarization illumination: filtration of *λ* = 620 nm with blue bars (*T*_*λ*=620_), visible light transmission with red bars (*T*_*λ*=700_), and yellow bars for IR blockage at *λ* = 2500 nm (Δ*T*_*λ*=2500_) of the structure with *p* = 800 nm, *w* = 700 nm, *θ* = 0 with different *m*.

To have a better insight, in Fig. 7 the bar chart of the three window parameters *T*_*λ*=620_, *T*_*λ*=700_, and Δ*T*_*λ*=2500_ of the studied structure in Fig. 6 with different WS_2_ layers *m* = 0, *m* = 1, *m* = 5, and *m* = 10 is plotted. Considering the data of Fig. 7 shows that insertion of one atomic layer of WS_2_ reduces the *T*_*λ*=620_ compared with that of the structure without WS_2_ but it is still more than that of a uniform VO_2_ window. By increasing the WS_2_ layer numbers to *m* = 5, *T*_*λ*=620_ reaches values not only less than the structure without WS_2_ but also less than the uniform VO_2_ layer. Increasing the number of WS_2_ layers to *m* = 10 decreases *T*_*λ*=620_ even more but at the cost of reduction of visible light transmission, *T*_*λ*=700_, and IR blockage, Δ*T*_*λ*=2500_. This way, to change the window color together with keeping IR blockage and visible light transmission as high as possible, in the structure with *p* = 800 nm and *w* = 700 nm, we select the number of WS_2_ layers as *m* = 5.

**Fig. 8:**
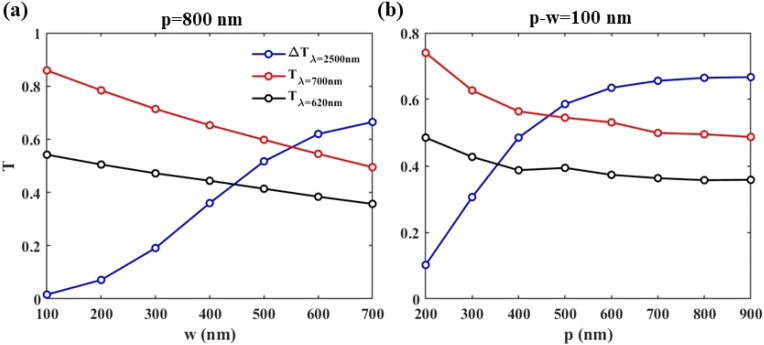
Δ*T*_*λ*=2500_ (blue circles), *T*_*λ*=700_ (red squares), and *T*_*λ*=620_ (black triangles) of the structure as a function of: (a) *w* with *p* = 800 nm and *m* = 5 and (b) *p* in structures with *p* − *w* = 100 nm and *m* = 5 when *p* = 800 nm and *w* = 700 nm, under TM polarization illumination.

To summarize the results, in Fig. 8 we present Δ*T*_*λ*=2500_ (blue circles), *T*_*λ*=700_ (red squares), and *T*_*λ*=620_ (black triangles) of the structures with different parameters: (a) *p* = 800 nm, *m* = 5, and various values of *w* ranging from *w* = 100 nm to *w* = 700 nm; (b) *p* − *w* = 100 nm, *m* = 5, and various values of *p* ranging from *p* = 200 nm to *p* = 900 nm and (c) *p* = 800 nm, *w* = 700 nm, and different numbers of WS_2_ layers ranging from *m* = 0 to *m* = 10.

Fig. 8a shows that increasing the width of the ribbons from *w* = 100 nm to *w* = 700 nm decreases *T*_*λ*=700_, which is undesirable. However, given the nearly zero Δ*T*_*λ*=2500_ at *w* = 100 nm, makes it a necessity to select wider ribbons. With *w* = 700 nm as the optimum structure, under TM polarized illumination, Δ*T*_*λ*=2500_ = 67%, *T*_*λ*=700_ = 50%, and *T*_*λ*=620_ = 34%. Under TE illumination, these values reach Δ*T*_*λ*=2500_ = 61%, *T*_*λ*=700_ = 45%, and *T*_*λ*=620_ = 30%.

In Fig. 8b, we study structures with different periods but with the restriction of *p* − *w* = 100 nm. It can be seen that for structures with periods less than 600 nm, IR blockage is less than that of uniform VO_2_ (62%), making such structures undesirable. Above *p* = 600 nm, Δ*T*_*λ*=2500_, *T*_*λ*=700_, and *T*_*λ*=620_ remain almost the same, with maximum values of Δ*T*_*λ*=2500_ = 67% and *T*_*λ*=700_ = 50%, and a minimum value of *T*_*λ*=620_ = 34% in the structure with *p* = 800 nm and *w* = 700 nm.

**Fig. 9:**
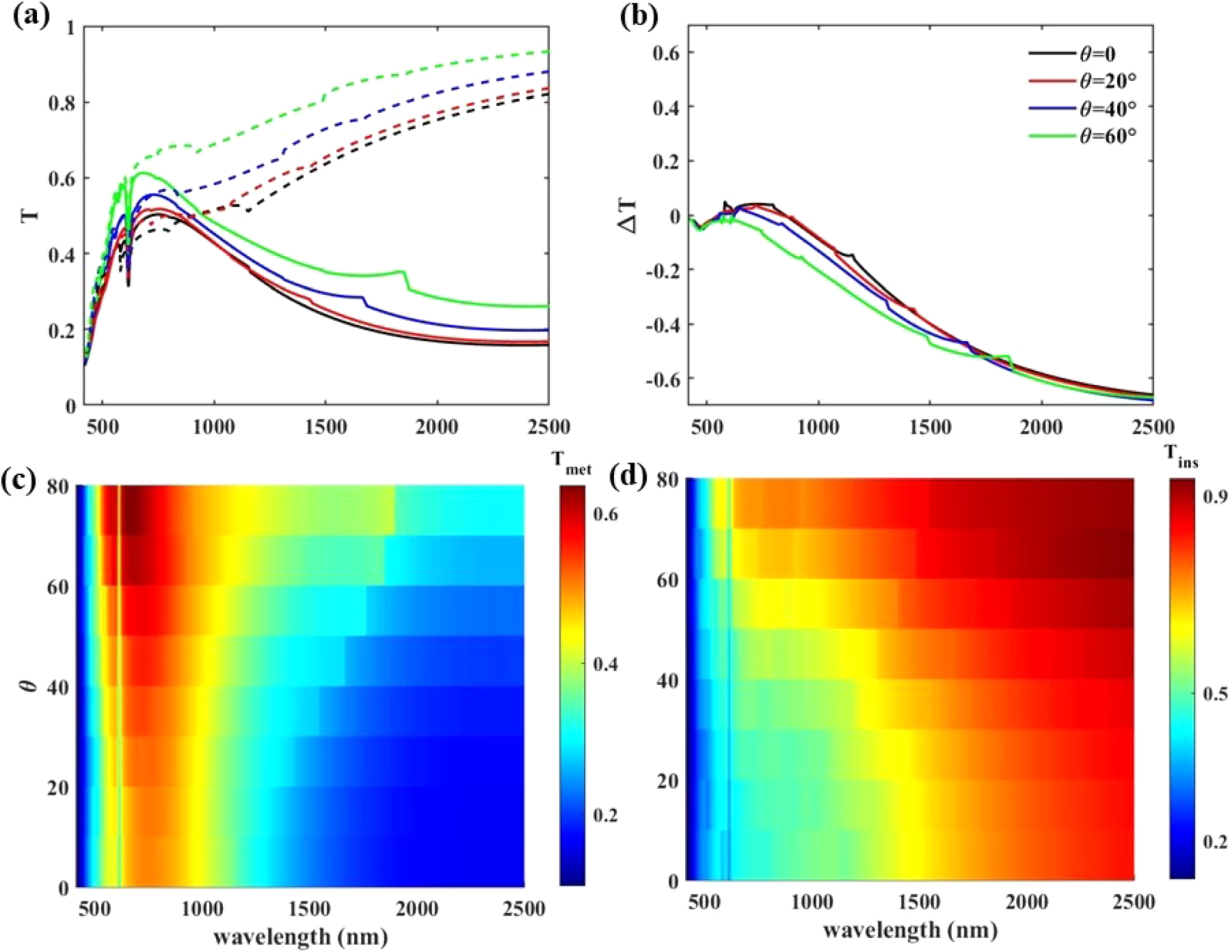
(a) *T* and (b) Δ*T* spectra of the structure with *p* = 800 nm, *w* = 700 nm, and *m* = 5 for different values of *θ* under TM polarization illumination. In (a) dashed and solid lines are for metallic and insulator states of VO_2_, respectively. To expand the range of *θ* values, transmission spectra of the structure when the VO_2_ is in its metallic and insulator state are included in (c and d), respectively.

As the angle of sunlight radiation varies during the day, it is necessary to test our designed window to see if it remains effective under different light illumination angles, *θ*. We investigate *T* and Δ*T* in Fig. 9a and b, respectively; for the structure with *p* = 800 nm, *w* = 700 nm, and *m* = 5 for different values of *θ*. Dashed and solid lines of Fig. 9a are for metallic and insulator states of VO_2_, respectively. It can be seen that, in both metallic and insulator states of VO_2_, changing *θ* keeps the observed dip at *λ* = 620 nm. Compared to the normal incidence, at *θ* = 60°, a maximum 8% change in Δ*T*_*λ*=700_ occurs, while this change is only 1.4% in case of *θ* = 20°; that is a small amount. To test the change in light transmission in Fig. 9b we include the Δ*T* spectrum. It is obvious that no drastic change in Δ*T* happens with changing *θ* from 0° to 60°. Therefore, visible light transmission, IR blockage, and the pleasant color of the window do not changing significantly within inclined light illumination. To extend the range of studied angles in Fig. 9c and d, the transmission spectra of the incident inclined light through the structure in the metallic and insulator states of VO_2_ are studied, respectively. As can be seen, the window keeps its functionality in different wavelength ranges under different illumination angles.

In Table 2 we compare *T*_vis_ and Δ*T*_IR_ that are reached with our proposed VO_2_ grating structure to some of other references that worked on these parameters in VO_2_-based structures. It can be seen that in designing smart windows different methods are used: doping,^58,59^ applying multilayer structures,^20,60^ and insertion of nanoparticles or coating them with VO_2_.^15,17,57^ Generally, in works utilizing doping methods, window color control and tuning the critical temperature of VO_2_ are prioritized, while the aim of works that used multilayer structures are the optimization of *T*_vis_ and Δ*T*_IR_. Consequently, in reports that work on window color we can’t see high values of *T*_vis_ and Δ*T*_IR_^58,59^ and in works that are working on improving *T*_vis_ and Δ*T*_IR_ the unpleasant color issue is not addressed. In our work, we optimize all three important factors of smart windows with *T*_vis_ = 52%, Δ*T*_IR_ = 67% and *T*_*λ*=620_ = 45% in the structure with *m* = 1 and *T*_vis_ = 0%, Δ*T*_IR_ = 67% and *T*_*λ*=620_ = 34% in the structure with *m* = 5 under TM polarized light illumination. Within the same geometry, under TE illumination, with *m* = 1, *T*_vis_ = 47%, Δ*T*_IR_ = 63% and *T*_*λ*=620_ = 38% and with *m* = 5, *T*_vis_ = 45%, Δ*T*_IR_ = 61% and *T*_*λ*=620_ = 30% are reached.

**Table 2:** Comparison of the reached *T*_vis_ and Δ*T*_IR_ from different works. *T*_l_ and *T*_h_ stand for the tested low and high temperatures that are below and above the critical temperature of VO_2_

Structure	Tuning parameter	*T*_vis_	Δ*T*_IR_	Color change	*T*_l_ (°C)	*T*_h_ (°C)	Year	Ref.
Self-templated VO_2_ film	*d*_VO_2__ = 10 nm	78%	29%	No	30	100	2024	18
	*d*_VO_2__ = 20 nm	78%	29%					
	*d*_VO_2__ = 50 nm	65%	54%					
	*d*_VO_2__ = 80 nm	49%	70%					
	*d*_VO_2__ = 120 nm	31%	58%					
	*d*_VO_2__ = 160 nm	25%	49%					
Multilayers of TiO_2_/VO_2_/TiO_2_	—	58%	50%	No	20	80	2003	20
TiN nanoparticles coated with VO_2_	—	58%	56%	No	20	80	2018	15
VO_2_ parabolic nanocone array	—	90%	4%	No	20	90	2024	57
Zn-doped VO_2_ V_1−*x*_Zn_*x*_O_2_ thin film	*x* = 0%	37%	65%	Yes	26	95	2013	58
	*x* = 0.038%	37%	58%					
	*x* = 0.077%	42%	45%					
Zr-doped VO_2_ foils	Zr-doping = 4.2%	57%	21%	Yes	25	90	2014	59
	Zr-doping = 8.5%	59%	22%					
	Zr-doping = 9.8%	63%	18%					
SiO_2_/VO_2_ core/shell 2D photonic crystal	*R*_core/shell_ = 400 nm	48%	66%	Yes	20	90	2016	17
	*R*_core/shell_ = 500 nm	41%	70%					
	*R*_core/shell_ = 600 nm	42%	71%					
	*R*_core/shell_ = 700 nm	60%	72%					
SiO_2_/VO_2_ bilayer films	*n*_SiO_2__ = 1.16	53%	48.5%	No	25	80	2018	60
	*n*_SiO_2__ = 1.34	64%	47%					
	*n*_SiO_2__ = 1.42	66%	48%					
VO_2_ grating on WS_2_/SiO_2_ (this work)	*m* = 1(TM/TE)	52%/47%	67%/63%	Yes	20	80		
	*m* = 5(TM/TE)	50%/45%	67%/61%					

## 4 Conclusion

In the context of smart windows, VO_2_ as a phase change material that transfers from insulator to metallic state above its critical temperature, plays an essential role. By changing the VO_2_ state to metal, less IR radiation can be transmitted through the window which is an advantageous. In the visible range of the spectrum, VO_2_ transmission in both insulator and metallic states is low, which means the loss of natural daylight inside the building. We showed that, by structuring the VO_2_ as a grating which stands on a silica substrate, not only has the issue of visible light transmission been solved, but also the IR blockage was increased. By utilizing gratings with *p* − *w* = 100 nm, with *p* values more than 600 nm, we reached the best values for visible light transmission and IR blockage. The other issue with VO_2_-based windows is the unpleasant yellow-brownish color of these windows. To fix this problem, we used atomic layers of WS_2_ in the structure. WS_2_, with its bandgaps in the visible range of the spectrum, increases the light absorption at *λ* = 620 nm that modifies the window color. In the structure with *p* = 800 nm, *w* = 700 nm, and *m* = 1, we reached Δ*T*_*λ*=2500_ = 67%/*T*_*λ*=2500_ = 63% and *T*_*λ*=700_ = 52%/*T*_*λ*=700_ = 47% under TM/TE illumination. If we compare these values with that of a uniform VO_2_ layer with Δ*T*_*λ*=2500_ = 62%, *T*_*λ*=700_ = 46%, and *T*_*λ*=620_ = 41%, it can be seen that within our proposed structure all the characteristic parameters of a smart window improve. We showed that, according to the CIE color system, our designed window, compared to the VO_2_ layer, lowered the brownish sense of the window color and causes a better perceived color of the window. To our knowledge, this study demonstrates for the first time that all three critical factors of visible light transmission, IR blockage, and pleasant window color are simultaneously optimized in VO_2_-based smart windows. We also showed that all window properties remain consistent under different light illumination angles. Our results offer a new path for efficient smart windows with optimal IR blockage, visible light transmission, and color.

## Conflicts of interest

There are no conflicts to declare.

## Data Availability

Data underlying the results presented in this paper are not publicly available at this time but may be obtained from the authors upon reasonable request.
